# Molecular Design Properties of OxyVita Hemoglobin, a New Generation Therapeutic Oxygen Carrier: A Review

**DOI:** 10.3390/jfb2040414

**Published:** 2011-12-16

**Authors:** John P. Harrington, Hanna Wollocko

**Affiliations:** 1 Department of Chemistry, State University of New York, New Paltz, NY 12561, USA; 2 OXYVITA, Inc., New Windsor, NY 12553, USA; E-Mail: hannaW@oxyvita.us

**Keywords:** acellular hemoglobin, zero-linked polymerization, hemoglobin-based-oxygen-carrier (HBOC)

## Abstract

OxyVita Hb is a new generation hemoglobin based oxygen carrier (HBOC) produced through modification of a zero-linked polymerization mechanism using activators which incorporate cross-linked bovine tetramer hemoglobin into “super-polymeric” macromolecules (Average molecular weight = 17 MDa) for the purpose of oxygen delivery when whole blood or packed red cells are not available. This molecular design approach was generated in order to address several fundamental biochemical and physiological weaknesses of previous generations of HBOCs. Observation during pre-clinical and clinical studies provided evidence that these early generation acellular HBOCs were directly associated with loss of retention within the circulatory system, extravasation across endothelial tissue membranes due to their small molecular size leading to arterial and venous vasoconstriction with coupled increases in mean arterial pressure (MAP). The inherent increase in molecular size and structural stability of the OxyVita Hb is a direct response to addressing these serious weaknesses that have occurred during the evolution of HBOC development within the past two decades. The nature of the zero-linked synthetic route eliminates any chemical linkers remaining in the product, eliminating side reaction concerns, such as reversibility and decomposition due to weak chemical bonds, dependency on temperature and pressure, and residual toxicity

## 1. Introduction

During the past several decades, extensive research efforts have been directed towards the molecular design of an alternate therapeutic oxygen carrier for use in the absence or as an alternative to whole blood or packed red blood cells for the delivery of oxygen in emergency or other clinical applications [1,2,3]. Most of these approaches have centered upon the development of cell-free (acellular) hemoglobin-based oxygen carriers (HBOC).

The rationale for considering the use of an acellular hemoglobin approach goes back many years and has its roots in nature as illustrated in both the terrestrial and marine environments [4,5]. In the course of natural evolution, the advantage of large polymeric acellular oxygen transport proteins was found to be effective for oxygen transport/delivery for many invertebrate organisms whether living within terrestrial or aquatic conditions. These large polymeric proteins all possess unique hierarchical quaternary structures composed of multiple heme-containing globins and usually involving non-heme linkers chains [6,7]. The most obvious characteristic is the lack of a cellular membrane associated with a mammalian red blood cell which normally houses a protective array of enzymes to maintain these hemoglobins in the reduced state necessary for functionality. These large oxygen carrying proteins include hemoglobins often referred to as erythrocruorins or chlorocruorins and hemocyanins (copper-oxygen binding) usually found in arthropods and crustaceans. Given these observations and our understanding of how these acellular oxygen delivery proteins function *in vivo*, the quest for the a safe and efficacious acellular HBOC for clinical applications has provided opportunities to pursue a variety of pathways in the molecular design to address the human physiological conditions under which they must function [8,9].

As one examines the efforts to address the development of a functioning HBOC for clinical use, there is implicit in this evolution a recognition that any proposed HBOC must function as an acellular molecular species within the human circulatory system. Unfortunately the details of how this was to occur did not receive adequate attention in the earlier generation of HBOCs, resulting in a series of physiological effects that were recognized to be detrimental for human clinical applications [9].

During the last several decades the evolution of HBOC development at the molecular level has seen important design considerations incorporated into new molecular species with the goal of addressing the many negative observed characteristics associated with the first and second generation HBOCs [10,11,12]. These design modifications were initiated with the introduction of an intermolecular cross-linking approach (HemAssist, M.wt. 64 kDa) reducing dimerization of the initial tetrameric hemoglobin. This was followed by several different polymeric approaches involving intermolecular cross-linking polymerization by glutaraldyhyde (a non-specific bifunctional cross-linking agent) to cross-link bovine hemoglobin tetramers producing a heterogeneous mixture of larger molecular weight species, (Hemopure, human application, and Oxyglobin, veterinary applications, M.wt. > 150 kDa), raffinose cross-linked human hemoglobin (Hemolink, M.wt. >100 kDa), and pyridoxylated glutaraldehyde cross-linked human hemoglobin (PolyHeme, M.wt. >120 kDa). Concurrently, several recombinant human hemoglobins were developed that allowed for modification of the oxygen binding properties of these recombinant hemoglobins (Optro, M.wt. 64 kDa).

These approaches eliminated the dimerization of stroma free hemoglobin (small molecular radii) associated with rapid elimination from the circulatory system and reduced stress on the glomerulus and kidneys. The larger molecular weight cross-linked human and bovine hemoglobins were designed with the advantage of increasing retention time within the circulation and limiting access to the smaller vascular membrane pores. The rationale here was to limit hemoglobin extravasation and mitigate nitric oxide NO binding that is now known to be associated with an increase in vasoconstriction coupled with an elevation in mean arterial pressure MAP [13]. Going in a different direction to alter the molecular size and hydrodynamic properties of an HBOC, the Hemospan product (selected pegylation, M.wt. ∼90 kDa) was introduced in the 1990′s to reduce extravasation thereby mitigating vasoconstriction and maintaining mean arterial pressure (MAP) [14]. This approach was associated with increased water of hydration leading to an increase in the effective hydrodynamic radius of these molecular species, reducing the tendency to extravasate. These different molecular design modifications resulted in several HBOCs with no observed dimerization and alleviated the initial clinical concern of rapid elimination from the circulation.

Although many of these early HBOCs employed in phase I to phase III studies received initial approval for clinical applications to demonstrate effectiveness in the delivery of oxygen in specific clinical situations, continued observations of oxidative and cellular damage associated with their use prevented full FDA regulatory approval for final clinical use [1,2]. The underlying reasons for this lack of approval by the FDA were due to the inadequacy of the fundamental chemistry design of several of these HBOCs and their physiochemical properties. Questions of molecular size and shape, structural integrity (conformational integrity and quaternary structural intactness), redox behavior and stability (ability to be maintained and function in the reduced state) within the human circulatory system are vital to an HBOC when functioning as a safe and efficacious therapeutic oxygen delivery system. Many earlier HBOC studies failed to provide this kind of structural information to the scientific community about the molecular integrity, stability, and redox activities of these modified HBOCs.

The focus of this review is to provide an essential level of information that is presently available about a new generation HBOC, OxyVita Hemoglobin. The design and development of OxyVita hemoglobin has resulted from evaluation of the lessons learned from the data or lack of data of many previous pre-clinical and clinical studies that have been carried out over the last several decades. The ultimate goal is to provide the clinical community with a safe and efficacious therapeutic oxygen carrier as an alternative to blood transfusions when blood or red blood cells are not available. The original effort in the development of this new generation HBOC began in the laboratory of Professor Enrico Bucci and his co-workers [14] at the University of Maryland, wherein they utilized a unique zero-linked hemoglobin polymerization technology. Further refinement of the OxyVita's molecular properties have been carried out within OXYVITA, Inc., who recognized that molecular size and the unique chemistry involved in the production of OxyVita hemoglobin were essential for the success of initial pre-clinical studies conducted by several independent investigators throughout the United States [15,16,17,18,19].

## 2. Experimental Development/Production of OxyVita Hemoglobin: Liquid/Powder Forms

### 2.1. Preparation of OxyVita Hemoglobin

Although any tetrameric mammalian hemoglobin may be used as the starting material for the preparation of a zero-linked polymeric hemoglobin, bovine blood was chosen as the raw material due to its ubiquitous availability world-wide. Fresh bovine blood is obtained from US Department of Agriculture approved facilities, providing appropriate documentation on the medical history of a herd. The purification of bovine hemoglobin is carried out through a process of red cell lysis using a phosphate buffer, pH 7.4, followed by a series of low-speed and high-speed centrifugation to remove cellular debris. The isolated hemoglobin is used immediately for the production of the polymerized OxyVita hemoglobin.

The initiation of the zero-linked polymerization process is governed by the use of a chemical “activator” that leads to the production of intermolecular polymers from the previously intramolecularly cross-linked bovine tetramers. In the production of OxyVita hemoglobin, the water soluble carbodiimide EDC [1-ethyl-3-(3-dimethylaminopropyl) carbodiimide] is used to activate the side chain carboxylate groups on the hemoglobin surface, resulting in a highly reactive and short-lived *O*-acylisourea derivative. This isourea by-product is soluble in an aqueous solution and is removed directly by dialysis. The complex is formed from the C-terminal and Glu and Asp side chain carboxylate groups. These activated species react with the side chain of the lysyl residues of an adjacent hemoglobin molecule to form a stable amide bond (covalent), referred to as a pseudo-peptide bond [20]. Some interference may occur during this activation process wherein the activated carboxylic groups may be hydrolyzed by water, reducing their reactivity with the available lysyl amino groups. To improve on the efficiency of the polymerization process, a two-step approach to enhance the yield of the amide bond formation was introduced. The introduction of *N*-hydroxysulfosuccinimide (sulfo-NHS) to the carbodiimide reaction resulted in the formation of an intermediate sulfo-NHS ester, which then reacted with the amino groups [21]. One advantage of this approach is the possibility of modulating the extent of polymerization by altering the relative amounts of sulfo-NHS and EDC within the reaction mixture. Control of the rate, time and concentration within the polymerization process allows for regulation of the average molecular weight sizes of an individual preparation.

An extensive description of this zero-linked polymerization process as applied to the production of the original “Zero-Linked Bovine hemoglobin” (ZLBvHb) as originally described and produced by Professor Enrico Bucci and co-workers can be found in reference [14]. Earlier pre-clinical studies carried out utilized these initial preparations which typically contained a heterogeneous distribution of high molecular weight species in the range of 25MDa [22].

After the initial development of this zero-linked polymeric hemoglobin and with the acquisition of the license to manufacture this HBOC, OXYVITA, Inc., modified some of the preparation procedures to produce a more homogeneous molecular weight polymer with an average M.wt. of 17 MDa. Using anion-exchange (DEAE) and size exclusion chromatography (Fractogel 20–40) purification and isolation of discrete molecular weight fractions were achieved for this zero-linked polymerization process. Recent pre-clinical studies [17,19,23] have used the OxyVita hemoglobin preparation in their experimental protocols.

### 2.2. Preparation of OxyVita Hemoglobin-Powder Formulation

The powder form of OxyVita Hb is produced from the lyophilization of the liquid form of the protein. Recent acquisition of a new Virtus, Inc. lyophilization instrument allows for the production of this product under carefully controlled processing. The new instrument has the capabilities of monitoring (CFR/211 compliant software) the essential steps associated within the freeze-drying process as well as automatically sealing of the product, thus reducing the chances of any endotoxin introduction during the course of the operation. In creating the powder form of OxyVita Hb, consideration has been given to the number of different formulations that include the need for proper buffering, essential electrolytes and osmolarity of the final product for infusion applications. Currently work is proceeding to improve re-constitution time (solubility) for rapid application within a variety of clinical situations.

## 3. Results and Discussion

### 3.1. Unique Chemistry

The differentiating structural and chemical properties of OxyVita Hb are due fundamentally to the unique chemical and physical methods incorporated into its production as described in the methods section. OxyVita Hb is synthesized through a polymerization reaction of purified (ββ)cross-linked tetrameric bovine hemoglobin using controlled activators which are removed after the initial phase of synthesis. This activation process is primarily associated with the carboxylic surface residues of the tetramers which allow for the formation of the “zero-linked” molecular species. Selected modulation of these reactions allows for specific lysine residue involvement due to the differential pKs exhibited by these residues at pH 6.7. This approach allows for the elimination of any chemical linkers remaining within the product, eliminating possible side chain reaction concerns, such as reversibility and decomposition due to weak chemical bonds, dependency on temperature and pressure, and residual toxicity.

This production method utilized by OXYVITA, Inc. allows for enhanced manufacturing and quality control resulting in sustained identical batch to batch preparations with a mean molecular weight of 17 MDa as determined by dynamic light scattering. No more than 5% methemoglobin is present in each preparation. Given the unique chemistry associated with the use of reaction activators, variation of concentration of components involved, time and temperature of polymerization, this distinct polymerization process allows for the production of a range of molecular weight molecules that may find different clinical applications in the future. In addition, this polymerization process allows for the use of any mammalian blood (tetrameric hemoglobin) as the starting raw material for a zero-linked polymeric hemoglobin.

### 3.2. Implications for Structure-Function Relationship of OxyVita Hb

These pseudo-peptide bonds initiated by the activiators between the tetrameric hemoglobin molecules within OxyVita Hb play a crucial role in the overall conformational stability of these large polymeric molecules. Structural integrity and biological functionality depend upon the integrity of both intramolecular and intermolecular bonds within the tetrameric units and between the multiple tetrameric units (∼1,000 tetramers/polymer) that go to form this “super-polymeric” HBOC. Given the inherent secondary and tertiary structure of bovine hemoglobin (∼75% α-helical) and the fact that each tetramer is cross-linked between the β-82 lysine residues, this formation of a large number of amide bonds between adjacent tetramers, clearly suggests that this large polymeric hemoglobin will possess increased conformational stability and be very resistant to molecular unfolding. This resistance to conformational unfolding ought to provide increased protection to the heme-iron moieties responsible for the transport and reversible binding of molecular oxygen within the circulatory system and oxygen delivery within the cellular/tissue environment.

**Figure 1:**
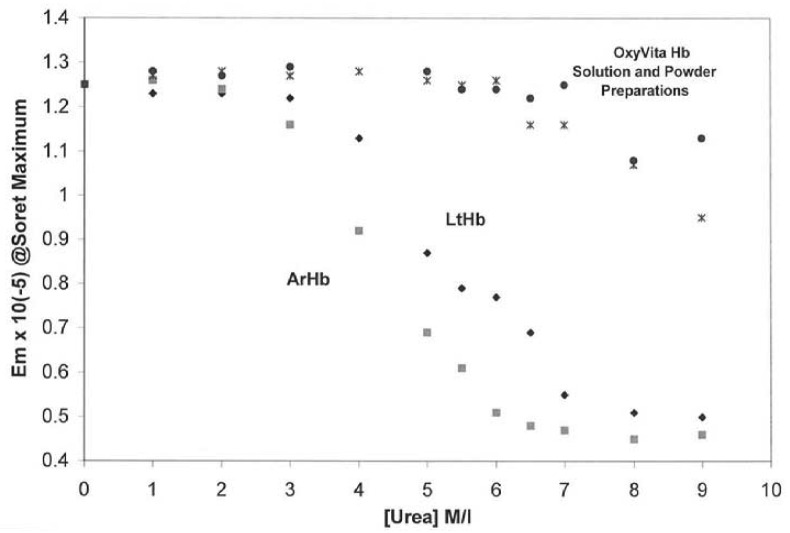
Isothermal Unfolding of Acellular Hemoglobins (*Arenicola* hemoglobin, ArHb; *Lumbricus* hemoglobin, LtHb; and OxyVita Hb, liquid (•) and powder (x) preparations in the presence of increasing concentrations of urea at T = 37 °C. All solutions were equilibrated for 30 min prior to spectral runs. Reference [24,25].

Studies on the structural integrity of this “super-polymeric” HBOC were carried out by isothermal unfolding studies at 37 °C using urea as the perturbant of the secondary and tertiary structure of these large molecules [24]. Figure 1 demonstrates the extraordinary resistance of this HBOC protein to molecular unfolding as compared to several natural acellular polymeric hemoglobins found in the terrestrial and marine environments. The Soret spectral region (350–450 nm) was used to determine the extent of secondary and tertiary conformational changes associated with this HBOC's unfolding due to its extreme sensitivity to alterations within the heme environment. When a hemoglobin molecule is in the presence of a conformational perturbant, such as urea, increasing its concentration leads to the disruption of many intramolecular interactions responsible for the maintenance of the integrity of the native functional structure.

**Figure 2:**
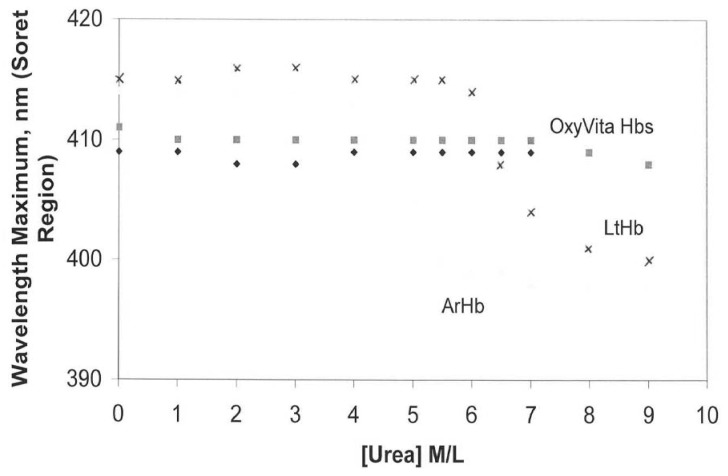
Changes in wavelength maximum within the Soret region for acellular hemoglobins in the presence of increasing concentrations of urea at T = 37 °C: OxyVita liquid (□), OxyVita powder (◆). Reference [24,25].

An additional advantage of using the Soret spectral region for evaluating conformational changes in hemoglobin is that it also allows for analysis of the redox state of the hemoglobin associated with the unfolding process. Maintenance of the heme-iron complex in the reduced state (heme-Fe^+2^) is essential for the reversible binding/release of molecular oxygen *in vivo*. As evident in Figure 2 changes in the oxidation state (heme-Fe^+2^ → heme-Fe^+3^) are reflected by a blue shift in wavelength maximum within the Soret region. In contrast to LtHb and ArHb, wherein the Soret wavelength maximum blue shifts are 413 nm → 400 nm and 412 nm → 398 nm, respectively, OxyVita Hb undergoes little change in the Soret maximum, 410 nm → 408 nm over the entire range of increasing urea concentrations. This significant blue shift, indicative of methemoglobin formation (heme-Fe^+3^), is associated with an increase in the extent of heme exposure during unfolding within these natural acellular hemoglobins [24,25,26,27]. Any increase in methemoglobin formation leads to a decrease in oxygen-carrying capacity.

All acellular hemoglobins exhibit varying amounts of methemoglobin formation via autoxidation with the potential for associated hemichrome formation and eventual release of the heme-iron which ultimately is responsible for cellular and tissue oxidative damage. Interestingly, a study by Jia and Alayash [23] clearly demonstrated that the zero-linked (OxyVita Hb) polymer gave no additional evidence of heme-iron loss compared to the initial bovine tetramers from which OxyVita Hb is constructed. This suggests that its heme stability is related to the well-defined compact conformational structure of this large polymer. These results are consistent with the observed resistance to the isothermal urea unfolding studies carried out as described above which demonstrate the inherent structural stability of OxyVita Hb as well as its reduced tendency to undergo oxidation in the presence of the denaturing perturbant [24].

A related observation on the redox behavior of OxyVita Hb is its ability to be reduced back to the oxyhemoglobin state in the presence of ascorbic acid, a known endogenous reducing agent found in the human plasma. This reaction has the potential to offer protection to the OxyVita Hb within the circulatory system without the benefit of the normal protective reducing enzymes that function within the red blood cell. Keeping the acellular OxyVita Hb in the reduced state will improve its functionality as an oxygen binding/release protein. Further studies in providing protection for this acellular polymeric hemoglobin are presently underway.

### 3.3. Effect of OxyVita Hb's Molecular Size on Other Physiological Functions

Table 1 presents the fundamental physicochemical characteristics of OxyVita Hb as determined by a wide range of biophysical methods. This polymeric hemoglobin has been successfully modified through selected chemical steps to produce the “super-polymer” referred to as OxyVita Hb possessing an average molecular weight of 17MDa and a hydrodymanic radius of 360 Å. The relative hydrodynamic viscosity for a 6 g% solution is 1.2 times higher than plasma and exhibits a colloidal osmotic pressure of 3 mm Hg (in lactated-Ringer, pH 7.4, 23 °C) approximately 1/10 that of plasma. It exhibits a P_50_ = 6 mm Hg with an n value (Hill Coefficient) ∼1. Less than 5% methemoglobin is present in the final product.

**Table 1:** Physicochemical Characteristics: OxyVita Hb.

Average Hydrodynamic Radius	=360 A
Intrinsic Viscosity [η]_c →0_	=4.2 mL/g
Average Molecular Weight	=17 MDa
Colloidal Oncotic Pressure	=3 mmHg
Viscosity at 6 g/dL	=similar to plasma
Intravascular retention time	>10 h (cats, n = 5)
Oxygen Affinity (P_50_)	=6 mmHg
Cooperativity (Hill)	=1.0–1.2
Met Hb	<5%

Further studies [15,25] using a wide range of physical and chemical methods over the last several years have clearly shown that the polymeric hemoglobin constituting the OxyVita Hb solution, the OxyVita Hb powder form and the Small Volume Resuscitation Fluid (SVRF) are equivalent in all their properties as evident in Table 2. The SVRF is composed of OxyVita Hb (9 g%) and NaCl (7.5 g%).

**Table 2:** Physicochemical Properties of OxyVita Hb.

	***OxyVita Solution***	***Small Volume Resuscitation Fluid***	***OxyVita Powder in Water***
Size Exclusion LC-POLYMERS	100%	99.2%	100%
Size Exclusion LC-TETRAMERS	0%	0.8%	0%
Spectral Ratio A_576_/A_540_	0.963	0.963	0.964
Autoxidation-MetHb	3.7%	3.0%	7.92%
Oxygen Affinity P_50_ a	5.95	5.26	6.59
Hill Coefficient (n value)	1.1	1.2	1.2
Dynamic Light Scattering b (average hydrodynamic radius)	360 Ǻ	365 Ǻ	359 Ǻ
Dynamic Light Scattering b (average molecular weight)	17,337 kDa	17,400 kDa	17,154 kDa
pH	7.46	7.50	7.58

a Hemox Analzyer (T = 37 °C, pH 7.50);

b Dyna-Pro 801 (Protein Solution) Reference [25].

The uniqueness of the chemical methods of synthesis of OxyVita Hb and its resultant molecular size have had profound impacts on some of the most important issues in the field of HBOC development. For years the question of loss of retention within the circulatory system associated with leakage of earlier HBOCs was associated with stress on the glomerulus and kidneys. More recently the nature of acellular HBOC extravasation and its impact on vasoconstriction and observed increase in mean arterial blood pressure (MAP) have been linked to complex interactions with nitric oxide (NO). During the past several years an understanding of these critical interactions has led to a better appreciation of the role of molecular size and its impact on the binding of NO and the concomitant physiological changes associated with any NO binding. The fact that increased molecular size can reduce the extent of acellular extravasation due to the molecules' inability to cross-over arterial or venous membranes which exhibit various pore sizes and bind with NO can lead to a reduction in vasoconstriction and help maintain MAP. In the case of OxyVita Hb the radius of this molecule allows for greater retention time in the circulatory system with a half-life of 8–12 times longer that smaller radius HBOCs. To date, all animal studies carried out with OxyVita Hb have resulted in no observable vascular extravasation as determined by its absence in the renal hilar lymph after exchange transfusion experiments [15]. Additional studies have shown no evidence of this acellular HBOC in the urine of these animal studies as evaluated by visible spectral analysis [28]. These observations are consistent with our present understanding of the complex nature of the mechanisms of intracellular extravasation through the endothelial capillary walls associated with a wide range of cellular pore sizes [29].

## 4. Summary

This review has addressed the reasons as to why OxyVita Hb, a new therapeutic oxygen carrier and HBOC, is fundamentally different from previous generations of HBOCs. As our understanding of the relationship between the structure and functional behavior of the earlier HBOCs evolved, it soon became apparent that many of the structural characteristics incorporated into the modified HBOCs still resulted in problems of extravasation and vasoconstriction, oxidative degradation, and safety concerns when these HBOCs were used in many pre-clinical and clinical testing studies. OxyVita Hb was designed to address these serious concerns and the fundamental aspects of OxyVita Hb's development and success to date within the earlier pre-clinical studies are based upon its unique chemical technology and the recognition that molecular size, hydrodynamic properties, negative surface charge [27] and discrete functional properties all matter in its physiological role as an HBOC. As described above, OxyVita Hb is differentiated from all other HBOCs presently being investigated due to: (1) its chemistry: a unique zero-linked polymerization method of synthesis; (2) well characterized physicochemical studies associated with its molecular properties and functional behavior; and (3) its potential for long-term storage under a wide range of climatic conditions due to its extraordinary molecular stability.
